# Interleukin-17a Induces Neuronal Differentiation of Induced-Pluripotent Stem Cell-Derived Neural Progenitors From Autistic and Control Subjects

**DOI:** 10.3389/fnins.2022.828646

**Published:** 2022-03-14

**Authors:** Ana Karolyne Santos Gomes, Rafaelly Mayara Dantas, Bruno Yukio Yokota, André Luiz Teles e Silva, Karina Griesi-Oliveira, Maria Rita Passos-Bueno, Andréa Laurato Sertié

**Affiliations:** ^1^Hospital Israelita Albert Einstein, Centro de Pesquisa Experimental, São Paulo, Brazil; ^2^Centro de Estudos do Genoma Humano e Células Tronco, Instituto de Biociências, Universidade de São Paulo, São Paulo, Brazil

**Keywords:** maternal immune activation, autism spectrum disorder, interleukin-17a, induced-pluripotent stem cell-derived neural progenitor cells, neuronal differentiation

## Abstract

Prenatal exposure to maternal immune activation (MIA) has been suggested to increase the probability of autism spectrum disorder (ASD). Recent evidence from animal studies indicates a key role for interleukin-17a (IL-17a) in promoting MIA-induced behavioral and brain abnormalities reminiscent of ASD. However, it is still unclear how IL-17a acts on the human developing brain and the cell types directly affected by IL-17a signaling. In this study, we used iPSC-derived neural progenitor cells (NPCs) from individuals with ASD of known and unknown genetic cause as well as from neurotypical controls to examine the effects of exogenous IL-17a on NPC proliferation, migration and neuronal differentiation, and whether IL-17a and genetic risk factors for ASD interact exacerbating alterations in NPC function. We observed that ASD and control NPCs endogenously express IL-17a receptor (IL17RA), and that IL-17a/IL17RA activation modulates downstream ERK1/2 and mTORC1 signaling pathways. Exogenous IL-17a did not induce abnormal proliferation and migration of ASD and control NPCs but, on the other hand, it significantly increased the expression of synaptic (Synaptophysin-1, Synapsin-1) and neuronal polarity (MAP2) proteins in these cells. Also, as we observed that ASD and control NPCs exhibited similar responses to exogenous IL-17a, it is possible that a more inflammatory environment containing other immune molecules besides IL-17a may be needed to trigger gene-environment interactions during neurodevelopment. In conclusion, our results suggest that exogenous IL-17a positively regulates the neuronal differentiation of human NPCs, which may disturb normal neuronal and synaptic development and contribute to MIA-related changes in brain function and behavior.

## Introduction

In addition to strong evidence for the genetic transmission of autism spectrum disorder (ASD), exposure to environmental risk factors during critical periods of brain development can also influence risk for ASD. Prenatal exposure to maternal immune activation (MIA) has been implicated as an environmental risk factor for ASD. Several epidemiological studies have suggested that exposure of fetuses to maternal infection during pregnancy increases the likelihood of developing ASD (Wilkerson et al., 2002; Atladóttir et al., 2010; Brown et al., 2014; Lee et al., 2015). In support to this notion, animal models have shown that MIA induced experimentally *via* immunogens (such as poly I:C, a synthetic double-stranded RNA to mimic viral infection) results in offspring with cortical malformations, behavioral symptoms, and immune dysfunctions reminiscent of ASD (Smith et al., 2007; Abrahams and Geschwind, 2010; Hsiao et al., 2012; Malkova et al., 2012; Choi et al., 2016; Lombardo et al., 2018).

Recent studies point to an important role for maternal interleukin-17a (IL-17a) in ASD-like phenotypes associated with MIA. IL-17a is a pro-inflammatory cytokine produced mainly by T helper 17 (Th17) lymphocytes that upregulates inflammatory gene expression usually associated with innate immune signaling and whose dysregulation may lead to chronic inflammation (Gaffen, 2009; Amatya et al., 2017; Milovanovic et al., 2020). In the MIA mouse model of ASD, poly I:C injection into pregnant dams on embryonic day 12.5 (E12.5) resulted in a strong increase of IL-17a expression in the maternal serum and placenta, as well as augmented IL-17a receptor subunit A (IL17RA) expression in the cortex of the fetal brain, leading to abnormal laminar cytoarchitecture both in fetal and adult brain and to ASD-related behaviors (Choi et al., 2016; Kim et al., 2017; Yim et al., 2017; Lammert et al., 2018). Importantly, similar pathological findings were produced by injecting recombinant IL-17a directly into the ventricles of the fetal brain at E14.5 in the absence of MIA (Choi et al., 2016). In addition, it was shown that chromic exposure of dams with IL-17a continuously throughout gestation also leads to neurodevelopmental and behavioral phenotypes relevant to ASD (Gumusoglu et al., 2020). Further, in humans, abnormal IL17A gene copy number variations (van der Zwaag et al., 2009) and elevated serum levels of IL-17a (Suzuki et al., 2011; Al-Ayadhi and Mostafa, 2012; Akintunde et al., 2015) were observed in ASD subjects.

Within the brain, IL17RA is expressed by various types of cells and previous *in vitro* and *in vivo* studies in mouse models have shown that IL17RA/IL-17a signaling exerts effects on neural progenitor cells (NPCs) (Li et al., 2013; Tfilin and Turgeman, 2019), neurons (Liu et al., 2014; Tfilin and Turgeman, 2019; de Lima et al., 2020), astrocytes (Kostic et al., 2017), oligodendrocytes (Wang et al., 2017), and microglia (Sasaki et al., 2020). However, the mechanisms by which IL-17a affects the human developing brain and the specific cell types that are affected by IL-17a signaling to contribute to ASD-related deficits are still poorly understood. Also, it remains to be determined whether IL-17a could synergize with genetic risk factors for ASD exacerbating brain abnormalities. Here, we used induced-pluripotent stem cell (iPSC)-derived NPCs from individuals with ASD of both known and unknown genetic cause as well as from neurotypical controls to address the direct effect of IL-17a on human NPC proliferation, migration and neuronal differentiation, and to investigate whether this cytokine can act in concert with underlying genetic susceptibilities altering NPC function.

## Materials and Methods

### Subjects and Genetic Analysis

All individuals evaluated in this study (*n* = 7 individuals with ASD and *n* = 5 neurotypical controls) were described previously (Sánchez-Sánchez et al., 2018; da Silva Montenegro et al., 2020; Griesi-Oliveira et al., 2021). CGH-array and whole exome sequencing using genomic DNA from the peripheral blood of the ASD subjects allowed the identification of known ASD pathogenic variants in three individuals: one carries a duplication of 17p13.3, the second carries a duplication of 15q11–13, and the third carries deleterious compound heterozygous variants in the *RELN* gene and a *de novo* splice site variant in the *CACNA1H* gene. The remaining four patients did not harbor rare variants (Global MAF ≤0.01 in gnomAD, 1000G, ESP6500, and AbraOM) that cause a deleterious loss of function of an ASD risk gene.^1^

### Neural Progenitor Cells Culture

All NPC samples used in this study have been previously differentiated from iPSCs (Sánchez-Sánchez et al., 2018; Griesi-Oliveira et al., 2021). NPCs derived from 1 iPSC clone of each individual with ASD of known and unknown genetic cause, and either 1 or 2 iPSC clones of the control subjects were used in all experiments described herein. NPCs were cultured on dishes coated with 10 μg/ml poly-L-ornithine (Sigma Aldrich) and 5 μg/ml laminin (Thermo Fisher Scientific) in NPC medium containing: DMEM/F12, 0.25X N2-supplement, 0.5X B27-supplement, 20 ng/ml FGF (Thermo Fischer Scientific), and 20 ng/ml EGF (Peprotech). The cell culture medium was changed every other day.

### Elisa Assay

To measure IL-17a protein levels in NPC culture supernatants, NPCs were cultured in NPC medium until 90% confluence in 60 mm tissue culture plates, and the supernatants were collected after 48 h cultures and centrifuged to remove cell debris. Cell-free supernatants (2.5 ml) were then transferred to a new conical tube, and the proteins in the supernatants were precipitated by adding ice-cold acetone (4:1) and resuspended in 250 μl of RIPA buffer containing protease inhibitors (10× concentrated supernatants). IL-17a protein levels were quantified by Human IL-17A SimpleStep ELISA Kit (ab216167, Abcam), following the manufacturer’s instructions. Optical density measurements were taken at 450 nm using the Glomax^®^ Discover Microplate Reader (Promega). All test samples were analyzed in duplicate and fell below the minimum detectable levels of the Elisa assay (1.56 pg/ml).

### Analysis of IL17RA Turnover and Interleukin-17a-Induced Intracellular Signaling Pathways

To investigate whether treatment of NPCs with IL-17a could alter the expression of IL17RA, NPC samples were seeded in 60 mm plates at a density of 1 × 10^6^ cells and were cultured in NPC medium containing either vehicle (ultrapure water) or human recombinant IL-17a (rhIL-17a) (Thermo Fischer Scientific) at 10 and 50 ng/ml for 48 h. The dosage of rhIL-17a and length of treatment were based on previous *in vitro* studies in mouse models examining the effect of exogenous IL-17a treatment on NPCs (Li et al., 2013), astrocytes (Kostic et al., 2017), and oligodendrocytes (Rodgers et al., 2015; Wang et al., 2017). To evaluate the effect of IL-17a on the activity of the ERK1/2, mTORC1, and NF-kB signaling pathways, NPC samples were seeded in 35 mm plates at a density of 3 × 10^5^ cells and were cultured in NPC medium until 80–90% confluence. The medium was then replaced with NPC medium containing either vehicle or rhIL-17a at 50 ng/ml, and cells were cultured for 5, 15, and 30 min. The experiments were repeated twice with similar results.

### Analysis of Cell Proliferation

To evaluate the effect of IL-17a on NPC proliferation, two sets of experiments were conducted. For the first experiment, NPC samples were seeded in 12-well plates at a density of 1.5 × 10^5^ cells per well and cultured in NPC medium containing either vehicle or 10 and 50 ng/ml of rhIL-17a, and then counted at 24 and 48 h using the trypan blue exclusion method and automated cell counting (Countess^®^ II FL Automated Cell Counter, Thermo Fisher Scientific). For the second experiment, NPC samples were seeded into 12-well plates at density of 2.5 × 10^5^ cells per well and cultured in NPC medium containing either vehicle, or 10 and 50 ng/ml of rhIL-17a, or 5 nM of rapamycin (used as control), or 50 ng/ml of rhIL-17a plus 5 nM of rapamycin. Cells were counted at 72 and 144 h using the trypan blue exclusion method and automated cell counting. Each experimental condition was performed in two replicate wells. The experiments were repeated at least twice with similar results.

### Analysis of Cell Migration (Scratch-Wound Healing Assay)

Neural progenitor cell samples were seeded in 96-well plates at a density of 3.5 × 10^4^ cells per well and cultured in NPC medium until confluence. The 96-well WoundMaker (Essen Bioscience) was used to generate a wound area in the monolayer of cells. The medium was then replaced by DMEM/F12 (without FGF and EGF) supplemented with either vehicle or 10 and 50 ng/ml of rhIL-17a, and wound closure was monitored for 24 h and quantified with the IncuCyte^®^ system (Essen Bioscience). Relative wound density was defined as cell density in the wound area expressed relative to the cell density outside of the wound area over time. Each experimental condition was performed in six replicate wells. The experiments were repeated at least twice with similar results.

### Differentiation of Neural Progenitor Cells Into Neurons

To evaluate the effect of IL-17a on neuronal differentiation of NPCs, two sets of experiments were conducted. NPCs were seeded into 6-well plates coated with 20 μg/ml poly-L-ornithine and 10 μg/mL laminin. For the first experiment, after reaching 50–60% confluence, cells were cultured in neuron differentiation medium [DMEM/F12, 0.5X N2, 1X B27, 1 μM of retinoic acid (Sigma-Aldrich)] containing either vehicle or 10 ng/ml of rhIL-17a for 14 days. For the second experiment, after reaching 50–60% confluence, cells were cultured in neuron differentiation medium containing either vehicle or 50 ng/ml of rhIL-17a for 21 days. In both experiments, the medium was changed every three days. Both experiments were repeated twice with similar results.

### RNA Extraction and Quantitative Real-Time PCR

Total RNA from each cell sample was extracted using the NucleoSpin RNA kit (Macherey-Nagel), following the manufacturer’s instructions. RNA quality and quantity were assessed using the NanoDrop 3300 Fluorospectrometer (Thermo Fisher Scientific). The reverse transcription of 1 μg of total RNA was performed with SuperScript III First-Strand Synthesis System (Thermo Fisher Scientific), and the qPCR reactions were carried out with 60 ng of cDNA and predesigned TaqMan gene expression assays (IL-17RA, Hs01056316_m1; IL17A, Hs00174383_m1–Thermo Fisher Scientific) in a QuantStudio 6 Flex Real-Time PCR System (Applied Biosystem). The expression levels of the target genes were normalized to the HMBS housekeeping gene (assay Hs00609293_g1). All qPCR samples were run in duplicate. Results are expressed as the mean fold change of the normalized gene expression relative to a calibrator sample using the comparative CT method (ΔΔCt method). The experiments were repeated twice with similar results.

### Protein Extraction and Immunoblotting

Total protein extracts from each cell sample were obtained using Ripa Buffer containing protease and phosphatase inhibitor cocktails (Sigma-Aldrich) and quantified using a BCA protein assay kit (Thermo Fisher Scientific) and the Glomax^®^ Discover Microplate Reader (Promega). For immunoblotting, total proteins (10–20 μg) were separated by SDS–PAGE (8–10%) and transferred to nitrocellulose membranes (GE HealthCare), which were then blocked and incubated overnight at 4°C with the following primary antibodies: anti-IL17RA (1:1,000, #12661; Cell Signaling Technology); anti-phospho-p44/42 MAPK (Erk1/2) (Thr202/Tyr204) (1:1,000, #9101; Cell Signaling Technology); anti-p44/42 MAPK (Erk1/2) (1:1,000, #9102; Cell Signaling Technology); anti-phospho-RPS6-S240/244 (1:2,500, #5364; Cell Signaling Technology), anti-phospho-NF-κB-p65 (Ser536) (1:1,000, #3033; Cell Signaling Technology); anti-NF-κB-p65 (1:1,000, #8242; Cell Signaling Technology); anti-SOX2 (1:1,000, AB5603; Millipore); anti-Nestin (1:1,000, MAB5326; Millipore); anti-Synaptophysin-1 (1:1,000, ab32127, Abcam); anti-Synapsin-1 (1:1,000, #5297; Cell Signaling Technology); anti-TUJ1 (1:2,000, MMS435P; Covance); anti-Homer1 (1:1,000, ab97593, Abcam); anti-MAP2 (1:1,000, M9942; Sigma-Aldrich); anti-GFAP (1:1,000, AB5804; Millipore); anti-βactin (1:10,000, A2228; Sigma Aldrich), for loading control. Detection was performed using horseradish peroxidase-coupled anti-rabbit or anti-mouse secondary antibodies (1:2,000, #7074 or #7072; Cell Signaling Technology), ECL substrate (Bio-Rad) and the ChemiDoc MP Imaging System (Bio-Rad). The intensity of the bands was determined by densitometry using The Image Lab Software (Bio-Rad). For analysis of constitutive IL17RA expression in ASD- and control-derived NPCs, total proteins from all cell samples were loaded in parallel on the same gel. For all the other immunoblots, total proteins from IL-17a-treated and vehicle-treated cells of each individual were loaded into adjacent wells on the gel, with each gel containing samples from ASD and control cells; the data are expressed as fold change in normalized protein expression in IL-17a-treated relative to vehicle-treated cells. Images of original western blots used in this study are presented in Supplementary Figure 1.

### Immunofluorescence

Neurons were seeded on cover slips pre-coated with 20 μg/ml poly-L-ornithine and 10 μg/ml laminin. Cell monolayers were fixed with 4% paraformaldehyde (Sigma Aldrich) and, after permeabilization with blocking buffer (5% donkey serum and 1% triton in PBS) at RT for 1 h, cells were incubated with primary antibodies diluted in blocking buffer overnight at 4°C. The primary antibodies used were: anti-Synapsin-1 (1:200, #5297; Cell Signaling Technology); anti-MAP2 (1:500, M9942; Sigma-Aldrich). After PBS washings, cells were incubated at RT for 1 h with secondary antibodies conjugated with AlexaFluor594 or AlexaFluor488 (Thermo Fisher Scientific; 1:400). Nuclei were counterstained with DAPI (VECTASHIELD, Vector laboratories). Fluorescence images were obtained using a Zeiss LSM 710 confocal microscope system (Carl Zeiss Meditec AG) and used as an initial qualitative test.

### Statistical Analysis

The data are expressed as medians with interquartile ranges. Differences between the ASD and Control groups were stablished using the Mann-Whitney *U*-test. Differences between the treatment groups (rhIL-17a vs. vehicle) were done using the Wilcoxon signed-rank test. *p*-values ≤ 0.05 were considered statistically significant. The statistical power calculation was conducted using the PASS (Power Analysis and Sample Size) software, and power values ≥0.80 were considered appropriate to reject the null hypothesis of zero correlation.

## Results

### Induced-Pluripotent Stem Cell-Derived Neural Progenitor Cells From Autism Spectrum Disorder and Control Subjects Express IL17RA and Respond to Exogenous Interleukin-17a

To investigate the direct effect of IL-17a on the biological properties of human NPCs, we used NPC samples derived from iPSCs of individuals with ASD (*n* = 7) and controls (*n* = 5). All individuals with ASD have been described previously (Sánchez-Sánchez et al., 2018; da Silva Montenegro et al., 2020; Griesi-Oliveira et al., 2021); three of them harbor ASD risk variants, while in the remaining four individuals no rare variants that cause a deleterious loss of function of an ASD gene were found (idiopathic ASD). In addition, all ASD- and control-derived NPC samples have recently been characterized by the expression of typical lineage-specific markers (Nestin, SOX1, and SOX2), as well as the ability to differentiate into neurons and astrocytes (Mansur et al., 2021).

**FIGURE 1:**
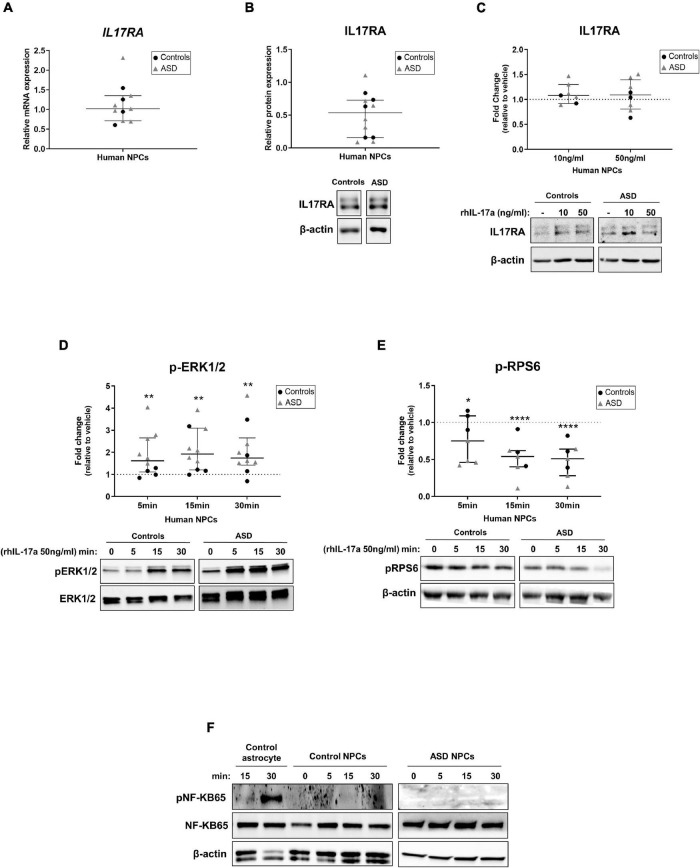
Human NPCs express IL17RA and respond to exogenous IL-17a by modulating the activity of ERK1/2 and mTORC1 signaling pathways. **(A)** Relative transcript levels of IL17RA in iPSC-derived NPCs of control (*n* = 4) and ASD (*n* = 7) subjects. **(B)** Relative protein levels of IL17RA in iPSC-derived NPCs of control (*n* = 5) and ASD (*n* = 7) subjects. β-actin was used as a loading control and representative immunoblot images are shown. Human NPCs endogenously express IL17RA. **(C)** Relative protein levels of IL17RA in iPSC-derived NPCs of control (*n* = 2–3) and ASD (*n* = 5) subjects after treatment with either vehicle (–) or rhIL-17a (10 and 50 ng/ml) for 48 h. The data are expressed as fold change in IL17RA expression relative to vehicle-treated samples. β-actin was used as a loading control and representative immunoblot images are shown. No significant differences in the protein levels of IL17RA were observed between rhIL-17a-treated and untreated human NPCs. **(D)** Time course analysis of pERK1/2 expression in iPSC-derived NPCs of control (*n* = 4) and ASD (*n* = 6) subjects after treatment with either vehicle (–) or rhIL-17a (50 ng/ml). Total-ERK was used as a loading control and the data are expressed as fold change in normalized pERK1/2 expression relative to vehicle-treated samples. Representative immunoblot images are shown. **(E)** Time course analysis of pRPS6 expression in iPSC-derived NPCs of control (*n* = 3) and ASD (*n* = 4) subjects after treatment with either vehicle (–) or rhIL-17a (50 ng/ml). β-actin was used as a loading control and the data are expressed as fold change in normalized pRPS6 expression relative to vehicle-treated. Representative immunoblot images are shown. Exogenous IL-17a led to a significant increase in pERK1/2 levels and significant decrease in pRPS6 levels in human NPCs. *p ≤ 0.05, **p ≤ 0.01, and ****p ≤ 0.0001. **(F)** Time course analysis of pNF-kB65 expression in iPSC-derived astrocytes (used as positive control of pNF-kB expression) and in iPSC-derived NPCs of control (*n* = 2) and ASD (*n* = 1) subjects after treatment with either vehicle (–) or rhIL-17a (50 ng/ml). Total-NF-kB and β-actin were used as a loading controls and representative immunoblot images are shown. While iPSC-derived astrocytes express pNF-kB65, no detectable expression of this protein was observed in human NPCs.

First, we measured the constitutive expression levels of IL-17a and IL17RA in ASD- and control-derived NPCs by RT-qPCR, Elisa and western blotting. While neither IL-17a mRNA nor IL-17a secreted protein were found in NPCs (data not shown), we observed that these cells express IL17RA mRNA and protein (Figures 1A,B). These results suggest that human NPCs endogenously express IL17RA, but not IL-17a under basal conditions.

To examine whether treatment of human NPCs with IL-17a alters the expression of IL17RA, ASD- and control-derived NPCs were cultured in NPC medium containing either vehicle or recombinant human IL-17a (rhIL-17a, 10 and 50 ng/ml) for 48 h, and the relative expression IL17RA was determined by western blotting. We did not observe significant differences in the protein expression levels of IL17RA between rhIL-17a-treated and untreated cells (Figure 1C), suggesting that, under these experimental conditions, exogenous IL-17a alone does not significantly affect endogenous expression of IL17RA in human NPCs.

We next sought to evaluate the effect of IL-17a on the activity of the ERK1/2 and mTORC1 signaling pathways, which regulate a wide variety of NPC processes (Wang et al., 2009; Paliouras et al., 2012; Hartman et al., 2013; Wyatt et al., 2014; Rhim et al., 2016), as well as of the NF-κB pathway, a classical downstream target of IL-17a that mediates immune and inflammatory responses (Gaffen, 2009; Amatya et al., 2017; Milovanovic et al., 2020), in ASD- and control-derived NPCs. Time course analysis showed that while treatment with rhIL-17a (50 ng/ml) led to a significant increase in phospho-ERK1/2 levels in NPCs (*p* ≤ 0.01) (Figure 1D), it induced a significant decrease in phospho-RPS6 levels, a downstream target of mTORC1 pathway, in these cells (*p* ≤ 0.05−*p* ≤ 0.0001) (Figure 1E). On the other, no detectable expression of phospho-NF-κB was observed in NPCs (Figure 1F). These results indicate that IL-17a alone does not activate the NF-KB pathway in human NPCs, whereas ERK1/2 and mTORC1 signaling pathways are regulated by IL-17a in these cells and, therefore, that this cytokine can exert a direct effect on human NPCs.

Finally, it is worth noting that no evident differences in the expression levels of IL17RA, phospho-ERK1/2, and phospho-RPS6 were found between ASD and control cells, but the sample size was insufficient to draw definitive conclusions about differences between the two groups (power <0.80).

### Interleukin-17a Has no Effect on the Proliferation and Migration Rates of Induced-Pluripotent Stem Cell-Derived Neural Progenitor Cells From Autism Spectrum Disorder and Control Subjects

**FIGURE 2:**
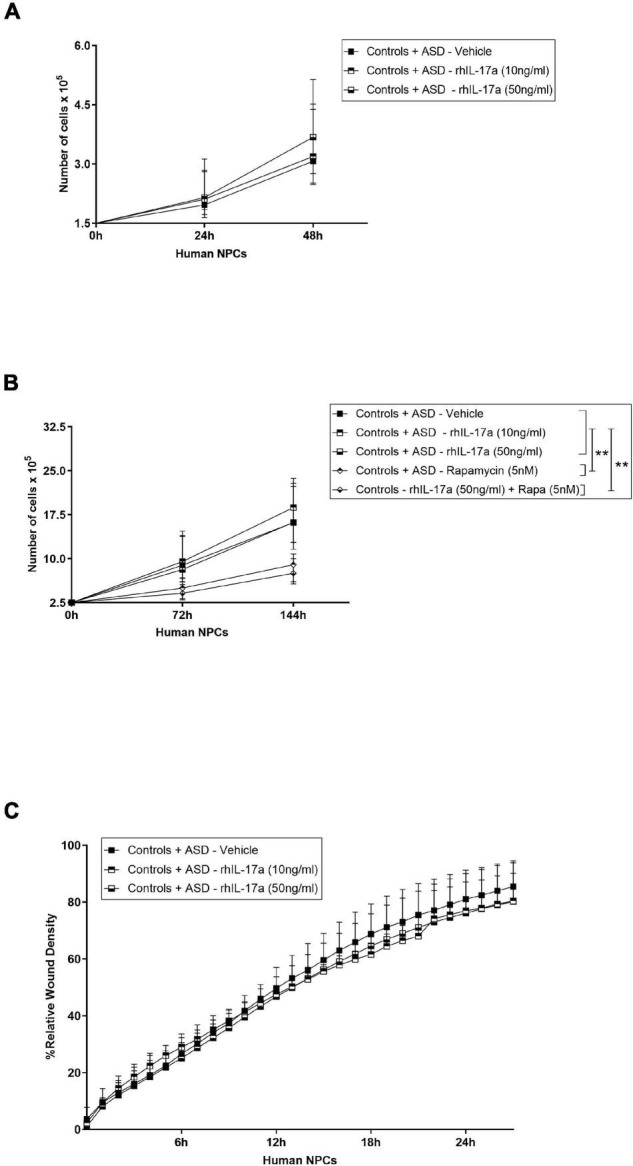
Exogenous IL-17a does not affect the proliferation and migration of human NPCs. **(A)** Line graph showing cell proliferation curves of iPSC-derived NPCs of control (*n* = 5) and ASD (*n* = 6) subjects cultured in the presence of either vehicle or rhIL-17a (10 and 50 ng/ml) for 24 and 48 h. **(B)** Line graph showing cell proliferation curves of iPSC-derived NPCs of control (*n* = 5) and ASD (*n* = 6) subjects cultured in the presence of either vehicle, or rhIL-17a (10 and 50 ng/ml), or rapamycin (5 nM, used as control), or rhIL-17a (50 ng/ml) plus rapamycin (5 nM) for 72 and 144 h. While rapamycin-treated human NPCs showed significantly decreased proliferation, **p ≤ 0.01, no significant differences were observed in the proliferation rates between rhIL-17a-treated and untreated human NPCs. **(C)** Line graph of mean% relative wound density over time in iPSC-derived NPCs of control (*n* = 5) and ASD (*n* = 3) subjects cultured in the presence of either vehicle or rhIL-17a (10 and 50 ng/ml) for 24 h. No significant differences were observed in the migration rates between rhIL-17a-treated and untreated human NPCs.

We next assessed the effect of IL-17a on the proliferation capacity of human NPCs. To this end, ASD- and control-derived NPCs were cultured in NPC medium containing either vehicle or rhIL-17a (10 and 50 ng/ml) for 24 and 48 h (Figure 2A), and then in NPC medium containing either vehicle, or rhIL-17a (10 and 50 ng/ml), or rapamycin (used as control), or rhIL-17a (50 ng/ml) plus rapamycin for an extended period of time, 72 and 144 h (Figure 2B). We observed that while treatment with either rapamycin alone or with rhIL17a plus rapamycin significantly decreased, as expected, the proliferation rate of NPCs (−*p* ≤ 0.01), rhIL-17a alone exerted no significant effect on the proliferation of these cells. Next, to investigate whether IL-17a alters the migration patterns of human NPCs, the scratch wound-healing assay was performed in ASD- and control-derived NPCs cultured in the presence of either vehicle or rhIL-17a (10 and 50 ng/ml). We observed that adding rhIL-17a did not significantly change the migration rates of the cells (Figure 2C). It is also noteworthy that no significant differences in proliferation and migration rates were observed between the ASD and control cells, but the sample size was not adequate to achieve statistical power and draw accurate conclusions about differences between the two groups. Taken together, these results suggest that exogenous IL-17a alone does not alter the proliferation and migration of human NPCs.

### Interleukin-17a Stimulates Neuronal Differentiation of Induced-Pluripotent Stem Cell-Derived Neural Progenitor Cells From Autism Spectrum Disorder and Control Subjects

**FIGURE 3:**
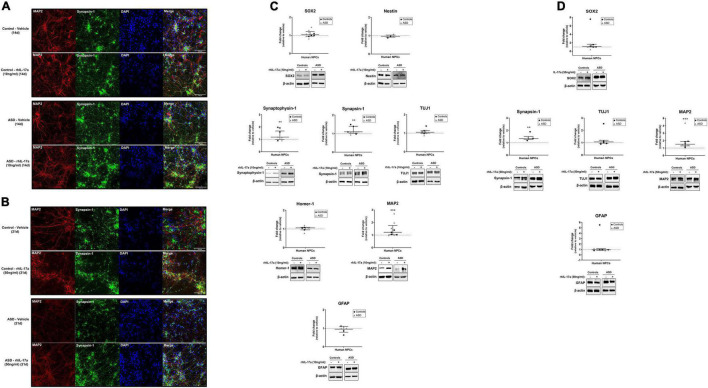
Exogenous IL-17a stimulates neuronal differentiation of human NPCs. **(A,B)** Representative images (20× objective) of qualitative immunocytochemistry staining of iPSC-derived neurons of control and ASD subjects differentiated in the presence of either vehicle or rhIL-17a (10 or 50 ng/ml) for either 14 or 21 day showing the expression of the synaptic protein Synapsin-1 and the cytoskeleton protein MAP2. Nuclei were stained with DAPI (blue). Control- and ASD-derived neurons show characteristic neuronal morphology, and no gross differences were observed between the control and ASD groups. **(C)** Relative protein levels of markers for NPCs (SOX2 and Nestin), neurons (Synaptophysin-1, Synapsin-1, TUJ1, Homer-1 and MAP2) and astrocytes (GFAP) in iPSC-derived neurons of control (*n* = 3–4) and ASD (*n* = 3–6) subjects differentiated in the presence of either vehicle (–) or rhIL-17a (10 ng/ml) for 14 day. β-actin was used as a loading control and the data are expressed as fold change in normalized protein expression relative to vehicle-treated samples. Representative immunoblot images are shown. **(D)** Relative protein levels of markers for NPCs (SOX2), neurons (Synapsin-1, TUJ1, and MAP2) and astrocytes (GFAP) in iPSC-derived neurons of control (*n* = 3–5) and ASD (*n* = 3) subjects differentiated in the presence of either vehicle (–) or rhIL-17a (50 ng/ml) for 21 day. β-actin was used as a loading control and the data are expressed as fold change in normalized protein expression relative to vehicle-treated samples. Representative immunoblot images are shown. While no significant differences were found in the expression levels of NPC and astrocyte markers between rhIL-17a-treated and untreated cells, significantly increased levels of several neuron markers were observed in rhIL-17a-treated human NPCs. **p ≤ 0.01, ***p ≤ 0.001.

Finally, we sought to determine whether IL-17a has any effect on the neuronal differentiation of human NPCs. For this purpose, ASD- and control-derived NPCs were cultured in neuronal differentiation medium containing either vehicle or rhIL-17a (10 and 50 ng/ml) for 14 days (Figure 3A) and 21 days (Figure 3B), and then the expression levels of typical markers for NPCs (SOX2 and Nestin), neurons (Synaptophysin-1, Synapsin-1, TUJ1, Homer and MAP2) and astrocytes (GFAP) were measured by western blotting (Figures 3C,D). While no significant differences were found in the expression levels of NPC and astrocyte markers between rhIL-17a-treated and untreated cells, we observed significantly increased levels (*p* ≤ 0.01−*p* ≤ 0.001) of several neuron markers (Synaptophysin-1, Synapsin-1, and MAP2) in rhIL-17a-treated NPCs. It should be noted that no significant differences in the expression levels of these neuronal proteins were observed between the ASD and control groups, but again the sample size was insufficient to reach definitive conclusions (power <0.80). Synaptophysin-1 and Synapsin-1 are neuron-specific proteins that regulate synaptic vesicle dynamics and neurotransmitter release in presynaptic terminals (Mirza and Zahid, 2018; Raja et al., 2019), and MAP2 is a neuron-specific cytoskeleton protein that stabilize microtubules regulating neuronal polarity and dendritic extension (Dehmelt and Halpain, 2005). Therefore, these results suggest that exogenous IL-17a positively regulates neuronal differentiation of human NPCs.

## Discussion

This study aimed to investigate whether IL-17a, a key pro-inflammatory cytokine associated with MIA-induced cortical and ASD-like behavioral abnormalities, affects human NPC biology and potentially synergizes with genetic risk factors for ASD changing NPC responses. NPCs are multipotent progenitor cells capable of extended self-renewal and with the ability to generate the three primary cell types of the central nervous system: neurons, astrocytes and oligodendrocytes (Anderson, 2001). Human iPSC-derived NPCs have been successfully used to model neurological diseases, including ASD (Mariani et al., 2015; Marchetto et al., 2017; Griesi-Oliveira et al., 2021), and we have recently shown that the transcriptome profiles from iPSC-derived NPCs of autistic and control subjects reflects neuronal tissue at early (4–10 post-conception weeks) stages of prenatal brain development (Griesi-Oliveira et al., 2021), corroborating that notion that these cells can reveal important clues about nervous system development and ASD pathophysiology.

Here we demonstrate that iPSC-derived NPCs from ASD and control subjects do not synthesize and secrete IL-17a but express IL17RA mRNA and protein. These findings are in accordance with previous studies in mouse models showing that astrocytes are the main local source of IL-17a under both physiological (Liu et al., 2014) and pathological (Lin et al., 2016) conditions, and that hippocampal NPCs express IL17RA (Li et al., 2013; Liu et al., 2014), which indicate that both local and systemic IL-17a can exert direct effects on these cells. We also found that treatment of ASD- and control-derived NPCs with rhIL-17a had no effect on the expression of IL17RA, which may suggest that undifferentiated NPCs are not the cellular source of upregulated IL17RA expression observed in the brain upon MIA (Choi et al., 2016). In line with this observation, a previous study has shown that Ank3- and NeuN-positive post-mitotic neurons, but not Pax6-positive neuronal progenitors, were the main cell population that expressed high levels of IL17Ra in the mouse fetal brain upon induction of MIA (Yim et al., 2017).

We observed that IL-17a/IL17RA signaling significantly activated the ERK1/2 pathway and inhibited the mTORC1 pathway in NPCs derived from ASD and control subjects. Although the functional consequences of IL-17a-mediated signaling thorough ERK1/2 and mTORC1 cascades for human NPCs are still unknown and deserve further investigation, it is noteworthy that previous studies have shown that IL-17a stimulates the differentiation of oligodendrocyte progenitor cells *via* activation of the ERK1/2 pathway (Rodgers et al., 2015), and enhances autophagy in ischemic neurons by inhibiting the mTORC1 pathway (Liu et al., 2019). Thus, our results suggest that ERK1/2 and mTORC1 pathways are downstream targets of IL-17a in human NPCs and may be associated with IL-17a-induced abnormal phenotypes of these cells.

Our results also showed that exogenous IL-17a alone had no effect on the proliferation of ASD- and control-derived NPCs. Studies in mouse models have provided controversial results on the effects of IL-17a on NPC proliferation. While some studies have reported that IL-17a negatively regulates the proliferation of mouse NPCs *in vitro* (Li et al., 2013; Tfilin and Turgeman, 2019) as well as of both adult-born neuroblasts and mature neurons in the hippocampus *in vivo* under physiological conditions (Liu et al., 2014), another study has suggested that this cytokine does not alter the proliferation of subventricular zone NPCs during stroke recovery, but instead increases their survival *in vivo* (Lin et al., 2016). Thus, taken together these findings suggest that the effect of IL-17a on NPC proliferation seems to be species and context dependent and needs to be explored further.

Administration of recombinant IL-17a to the fetal mouse brain has been shown to cause abnormal laminar cytoarchitecture (Choi et al., 2016), which suggests that an abnormal high level of this cytokine may exert detrimental effects on the migration of brain cells during neurodevelopment. However, the specific cellular populations with IL-17a-induced abnormal migration are still poorly explored. We observed that exogenous IL-17a alone had no effect on the migration of ASD- and control-derived NPCs under our experimental conditions, and thus it is tempting to speculate that migratory neuroblasts, mature neurons and/or glial cells, but not undifferentiated NPCs, may be the main targets of IL-17a during this pathological process. Interestingly, it has also been shown that intraventricular administration of IL-17a to mouse embryos led to abnormal migration and activation of cortical microglia (Sasaki et al., 2020), suggesting that these immune cells may also be involved in IL-17a-induced neuropathology.

Finally, our findings indicate that IL-17a has a significant positive effect on the neuronal differentiation of NPCs from ASD and control subjects. Few studies have examined the impact of IL-17a on neuronal differentiation, and the results are also context-dependent. It was shown that IL-17a knockout mice exhibited enhanced neurogenesis and neuronal excitability in the hippocampus, as well as that IL-17a deficient NPCs showed increased differentiation toward neurons *in vitro*, suggesting that IL-17a plays a negative role in regulating neurogenesis and neuronal differentiation under physiological conditions (Liu et al., 2014). On the other hand, it was also reported that treatment of mouse NPCs with recombinant IL-17a stimulated neuronal differentiation (Lin et al., 2016) and enhanced MAP2-positive neurite outgrowth (Tfilin and Turgeman, 2019) *in vitro*, which is in accordance with our findings. Interestingly, this effect of exogenous IL-17a on neurite outgrowth has also been reported for adult sympathetic post-ganglionic neurons (Chisholm et al., 2012) and adult dorsal root ganglia sensory neurons (Habash et al., 2015).

It is also noteworthy that, although a larger sample size would be necessary in order to have sufficient statistical power and effectively draw conclusions, we did not observe gross differences between ASD- and control-derived NPCs in response to IL-17a treatment, which may suggest that the genetic component of susceptibility to ASD did not change the effect of exogenous IL-17a in the proliferation, migration and neuronal differentiation of human NPCs under our experimental conditions. Previous studies have shown interactive effects of MIA and genetic risk factors for ASD in mouse models of tuberous sclerosis complex (Ehninger et al., 2012) and PTEN-associated autistic behavior and macrocephaly (Le Belle et al., 2014). Interestingly, the more pronounced brain overgrowth observed in the PTEN-mutant offspring was in part due to enhanced proliferation of NPCs (Le Belle et al., 2014). Therefore, it is possible that an inflammatory environment containing biomolecules besides IL-17a may be required to trigger gene-environment interactions during neurodevelopment of autistic children.

In conclusion, our results suggest that exogenous IL-17a stimulates the neuronal differentiation of human NPCs, which may disturb normal neuronal and synaptic development and may be involved with MIA-induced brain and behavioral changes.

## Data Availability Statement

The original contributions presented in the study are included in the article/Supplementary Material, further inquiries can be directed to the corresponding author.

## Ethics Statement

The studies involving human participants were reviewed and approved by The Ethics Committees of the Instituto de Biociências da Universidade de São Paulo and of the Hospital Israelita Albert Einstein approved this study, and written informed consent was obtained from all subjects’ caregivers.

## Author Contributions

AG, RD, BY, and ASi conducted the experiments and contributed to the acquisition, analysis, and interpretation of the data. AG wrote the first draft of the manuscript and prepared all figures with input from all authors. KG-O and MP-B critically reviewed the manuscript. ASe conceived the project, handled the funding, supervised the results, and edited the manuscript. All authors have read and agreed to the published version of the manuscript.

## Conflict of Interest

The authors declare that the research was conducted in the absence of any commercial or financial relationships that could be construed as a potential conflict of interest.

## Publisher’s Note

All claims expressed in this article are solely those of the authors and do not necessarily represent those of their affiliated organizations, or those of the publisher, the editors and the reviewers. Any product that may be evaluated in this article, or claim that may be made by its manufacturer, is not guaranteed or endorsed by the publisher.

## Footnotes

1
https://gene.sfari.org/

## Supplementary Material

Supplementary Figure 1 Original western blot images used in this study. **(A)** Original images of western blots showing expression levels of IL17RA in iPSC-derived NPCs of control (C; *n* = 5) and ASD (P; *n* = 7) subjects. β-actin was used as a loading control. **(B)** Original images of western blots showing expression levels of IL17RA in iPSC-derived NPCs of control (C; *n* = 2–3) and ASD (P; *n* = 5) subjects after treatment with either vehicle (−) or rhIL-17a (10 and 50 ng/ml) for 48 h. β-actin was used as a loading control. **(C)** Original images of western blots showing expression levels of pERK1/2 in iPSC-derived NPCs of control (C; *n* = 4) and ASD (P; *n* = 6) subjects after treatment with either vehicle (−) or rhIL-17a (50 ng/ml). Total-ERK was used as a loading control. **(D)** Original images of western blots showing expression levels of pRPS6 in iPSC-derived NPCs of control (C; *n* = 3) and ASD (P; *n* = 4) subjects after treatment with either vehicle (−) or rhIL-17a (50 ng/ml). β-actin was used as a loading control. **(E)** Original images of western blots showing expression levels of pNF-kB65 in iPSC-derived astrocytes (used as positive control of pNF-kB expression) and in iPSC-derived NPCs of control (C; *n* = 2) and ASD (P; *n* = 1) subjects after treatment with either vehicle (−) or rhIL-17a (50 ng/ml). Total-NF-kB and β-actin were used as a loading controls. **(F–M)** Original images of western blots showing expression levels of markers for NPCs (SOX2 and Nestin), neurons (Synaptophysin-1, Synapsin-1, TUJ1, Homer-1 and MAP2) and astrocytes (GFAP) in iPSC-derived neurons of control (C; *n* = 3–4) and ASD (P; *n* = 3–6) subjects differentiated in the presence of either vehicle (−) or rhIL-17a (10 ng/ml) for 14 day. β-actin was used as a loading control. **(N–R)** Original images of western blots showing expression levels of markers for NPCs (SOX2), neurons (Synapsin-1, TUJ1, and MAP2) and astrocytes (GFAP) in iPSC-derived neurons of control (C; *n* = 3–5) and ASD (P; *n* = 3) subjects differentiated in the presence of either vehicle (−) or rhIL-17a (50 ng/ml) for 21 day. β-actin was used as a loading control.
