# Supplementary figures and images for: Interleukin-17a Induces Neuronal Differentiation of Induced-Pluripotent Stem Cell-Derived Neural Progenitors From Autistic and Control Subjects
Source: Front Neurosci. 2022 Mar 14;16:828646. doi: 10.3389/fnins.2022.828646 (PMC8964130; doi:10.3389/fnins.2022.828646)

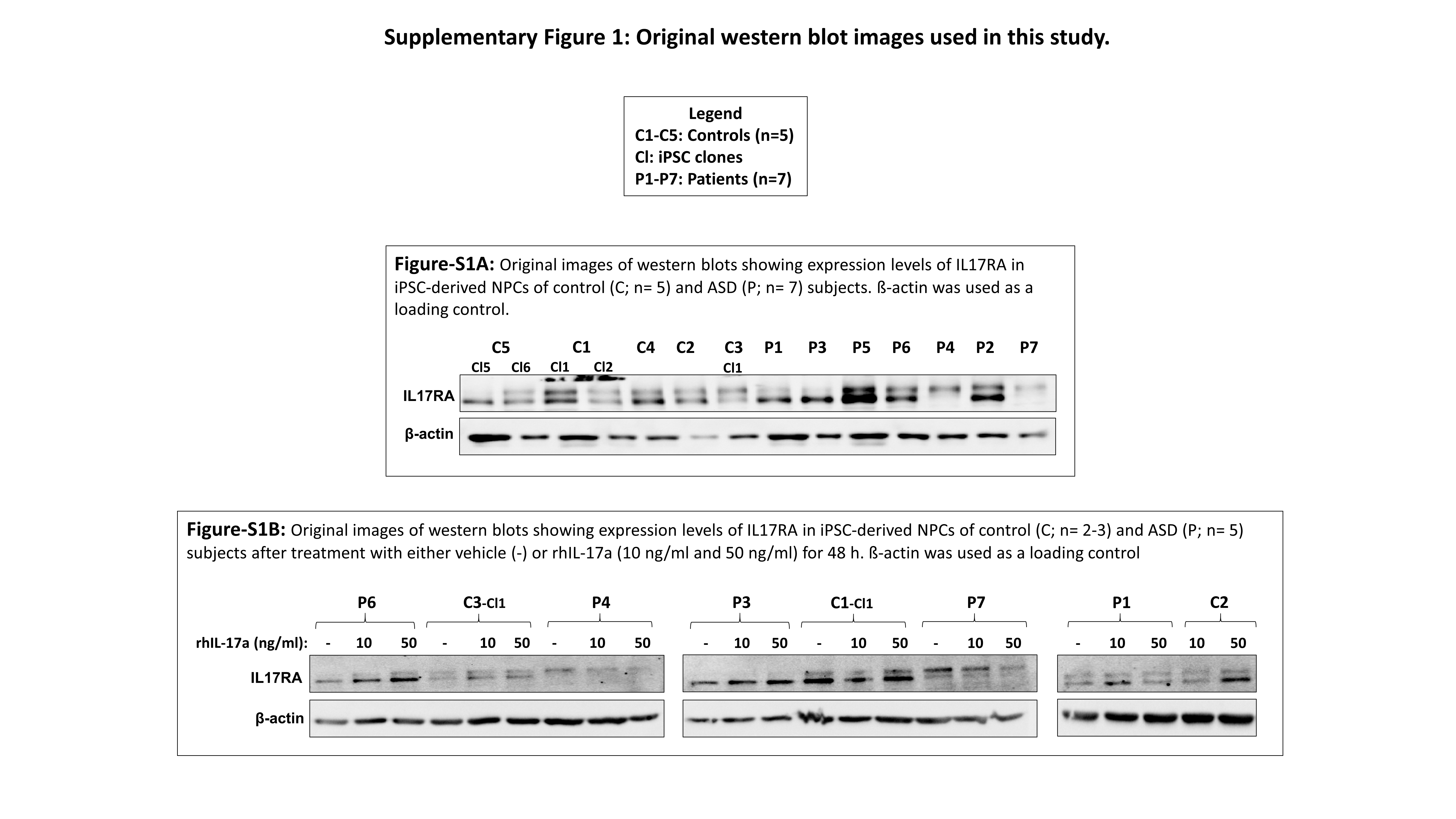

Supplement: Supplementary file 1 [file Image_1.TIFF]

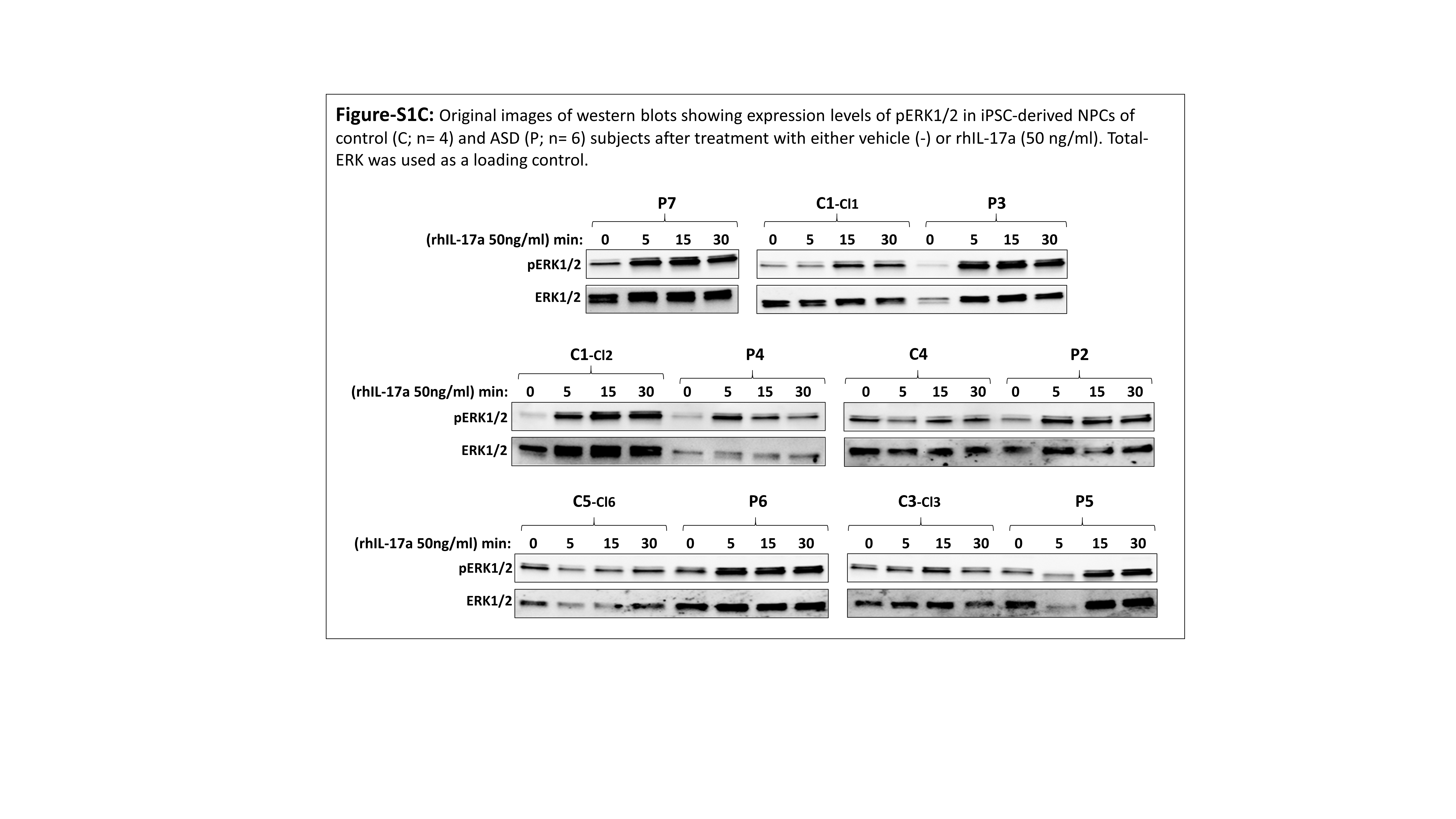

Supplement: Supplementary file 2 [file Image_2.TIFF]

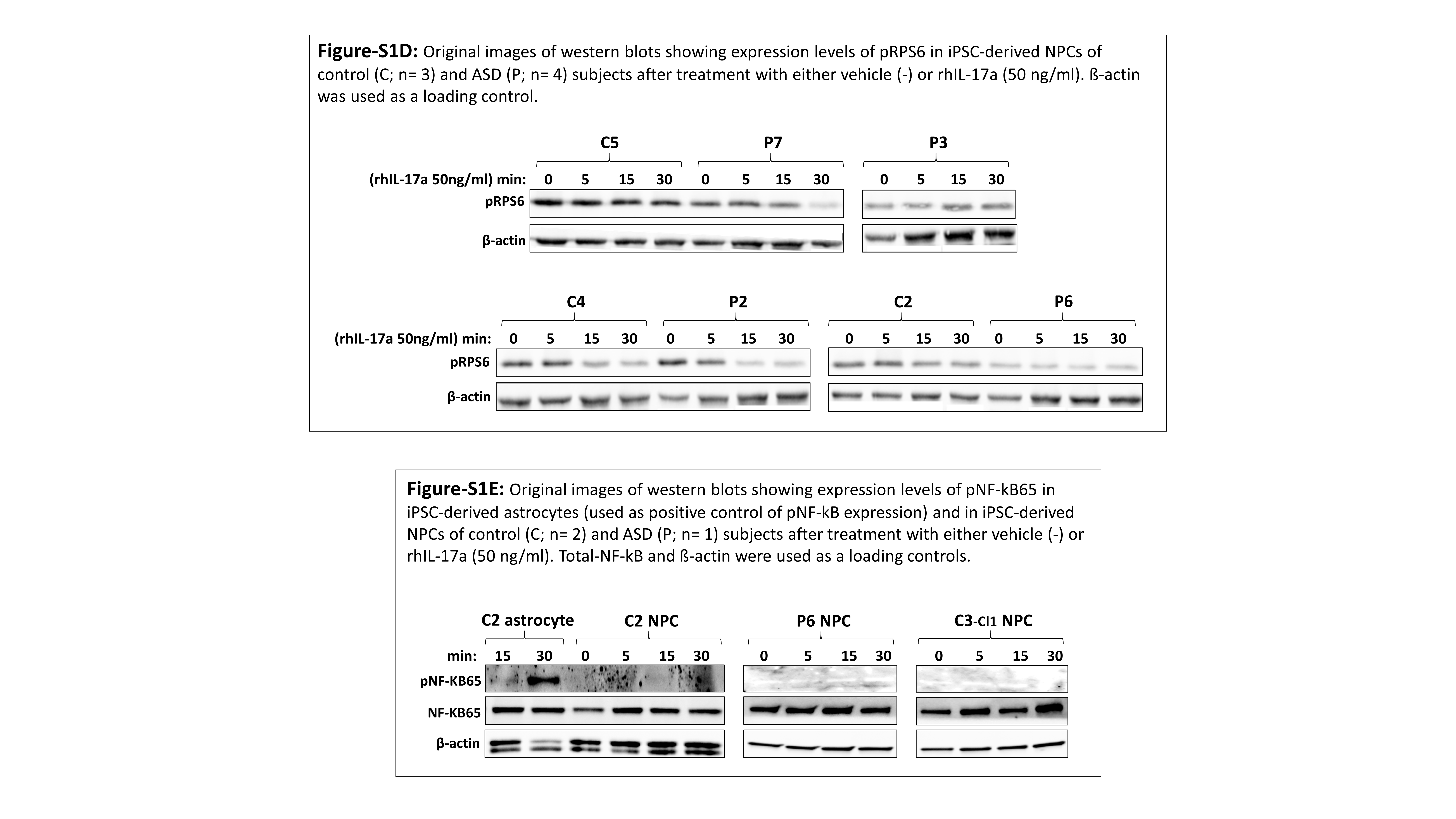

Supplement: Supplementary file 3 [file Image_3.TIFF]

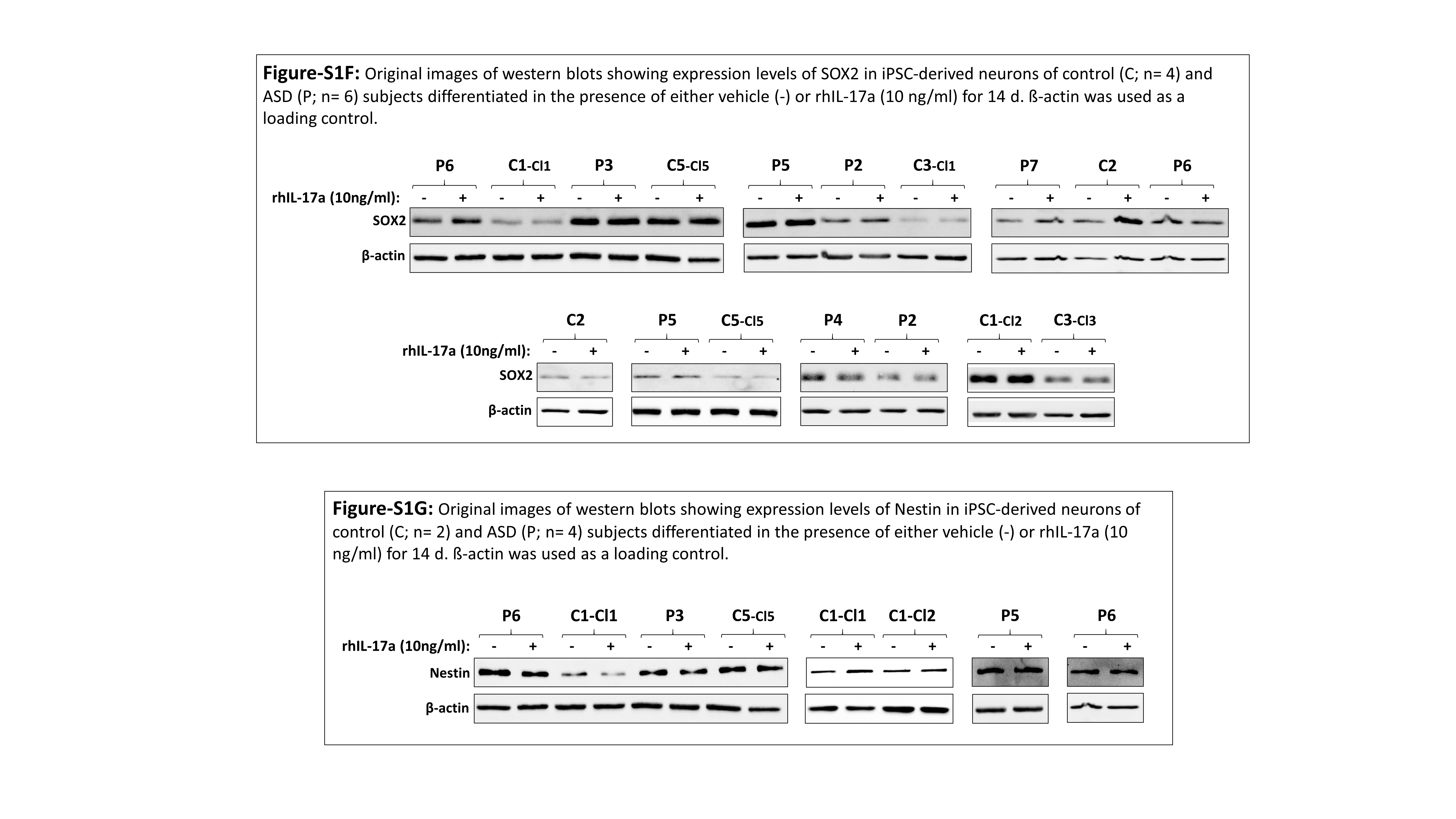

Supplement: Supplementary file 4 [file Image_4.TIFF]

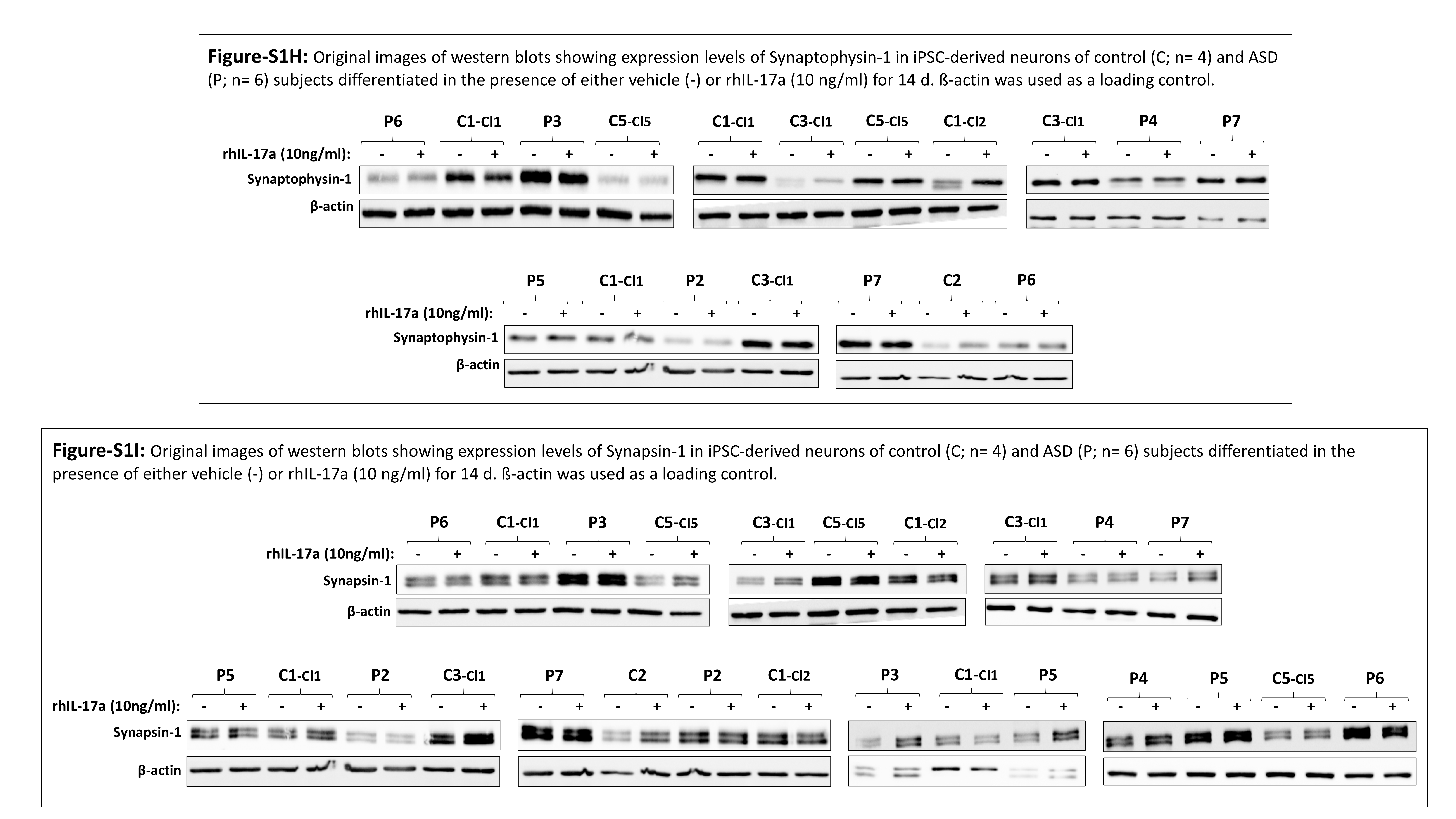

Supplement: Supplementary file 5 [file Image_5.TIFF]

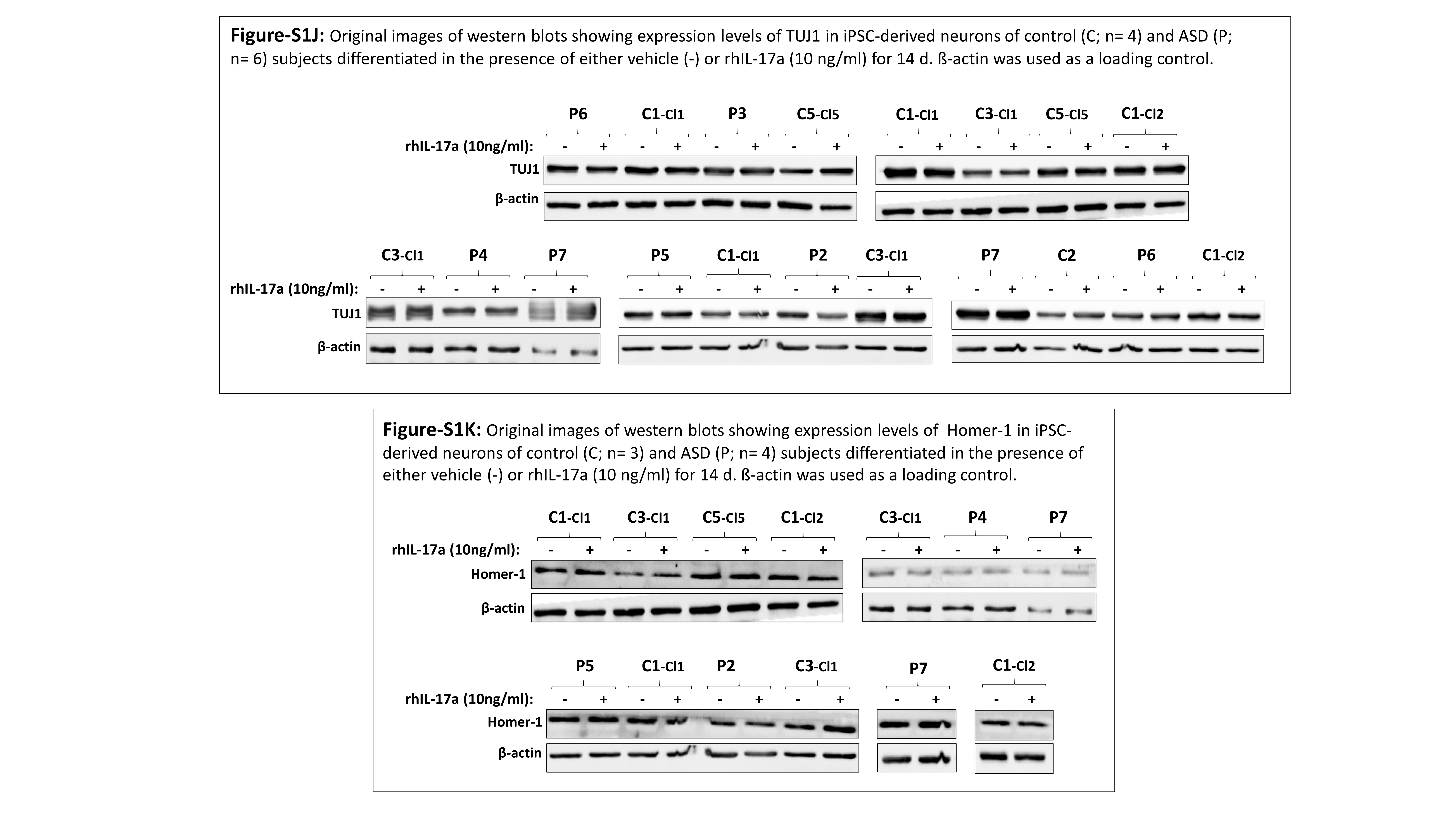

Supplement: Supplementary file 6 [file Image_6.TIFF]

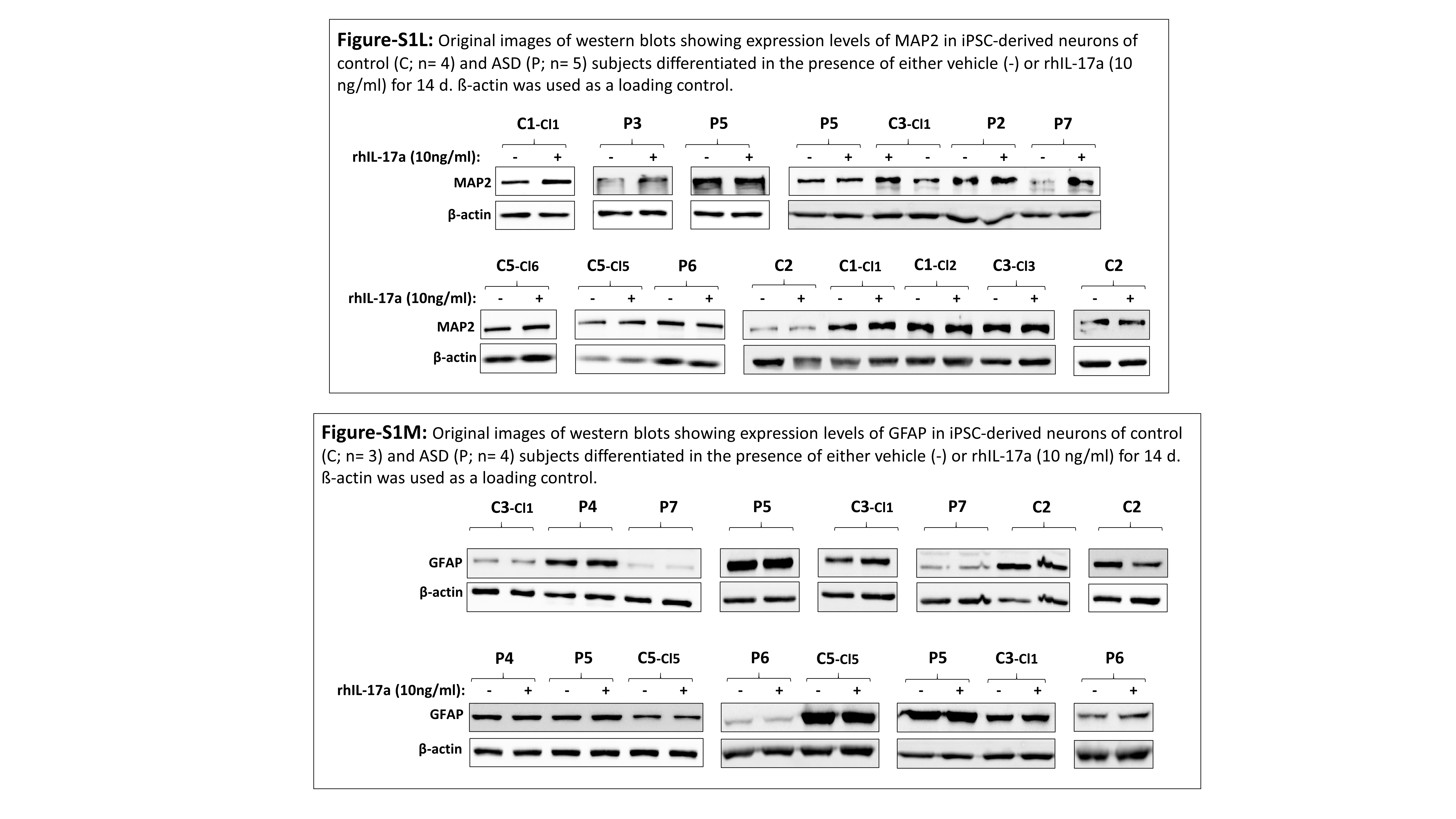

Supplement: Supplementary file 7 [file Image_7.TIFF]

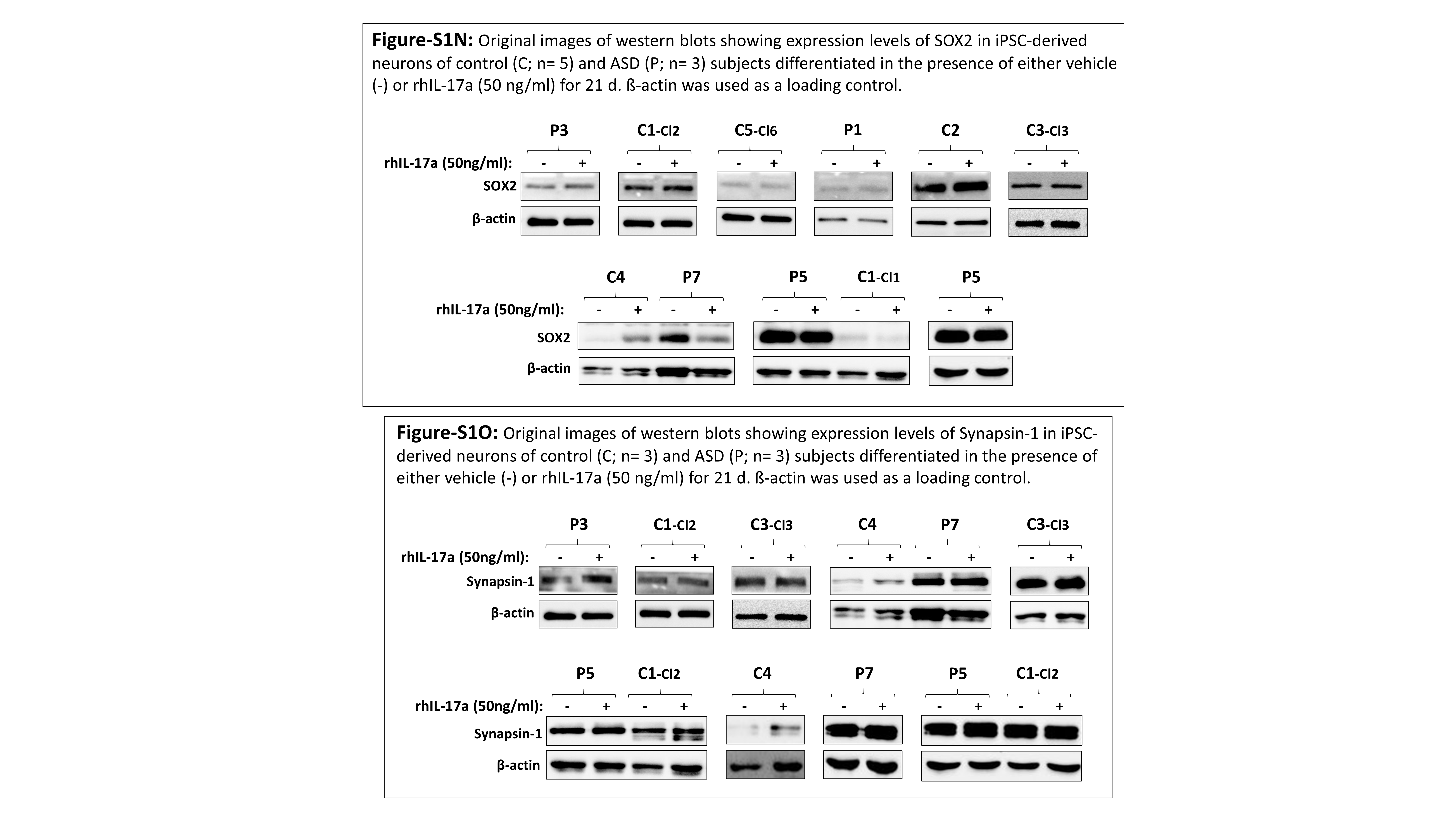

Supplement: Supplementary file 8 [file Image_8.TIFF]

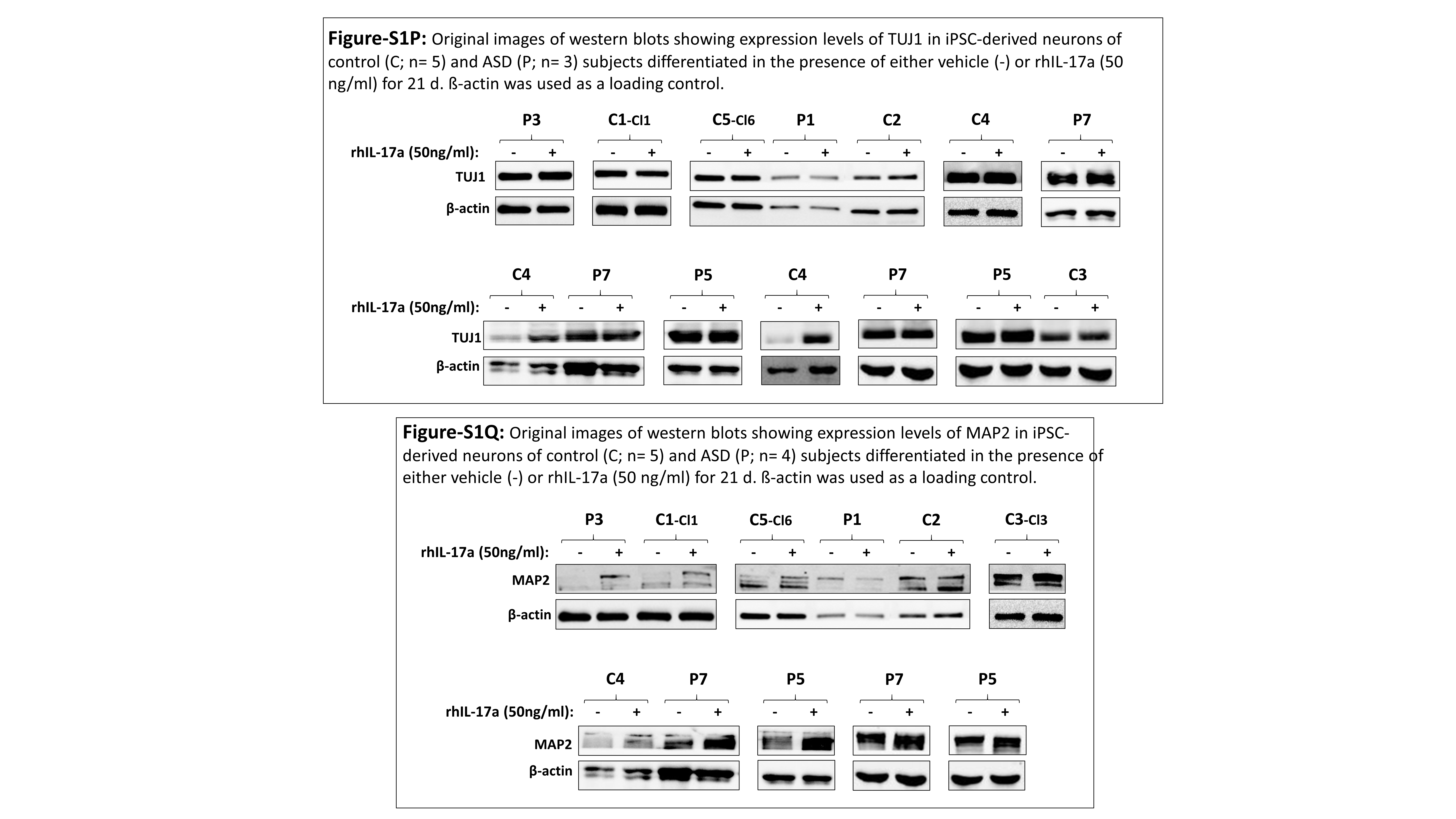

Supplement: Supplementary file 9 [file Image_9.TIFF]

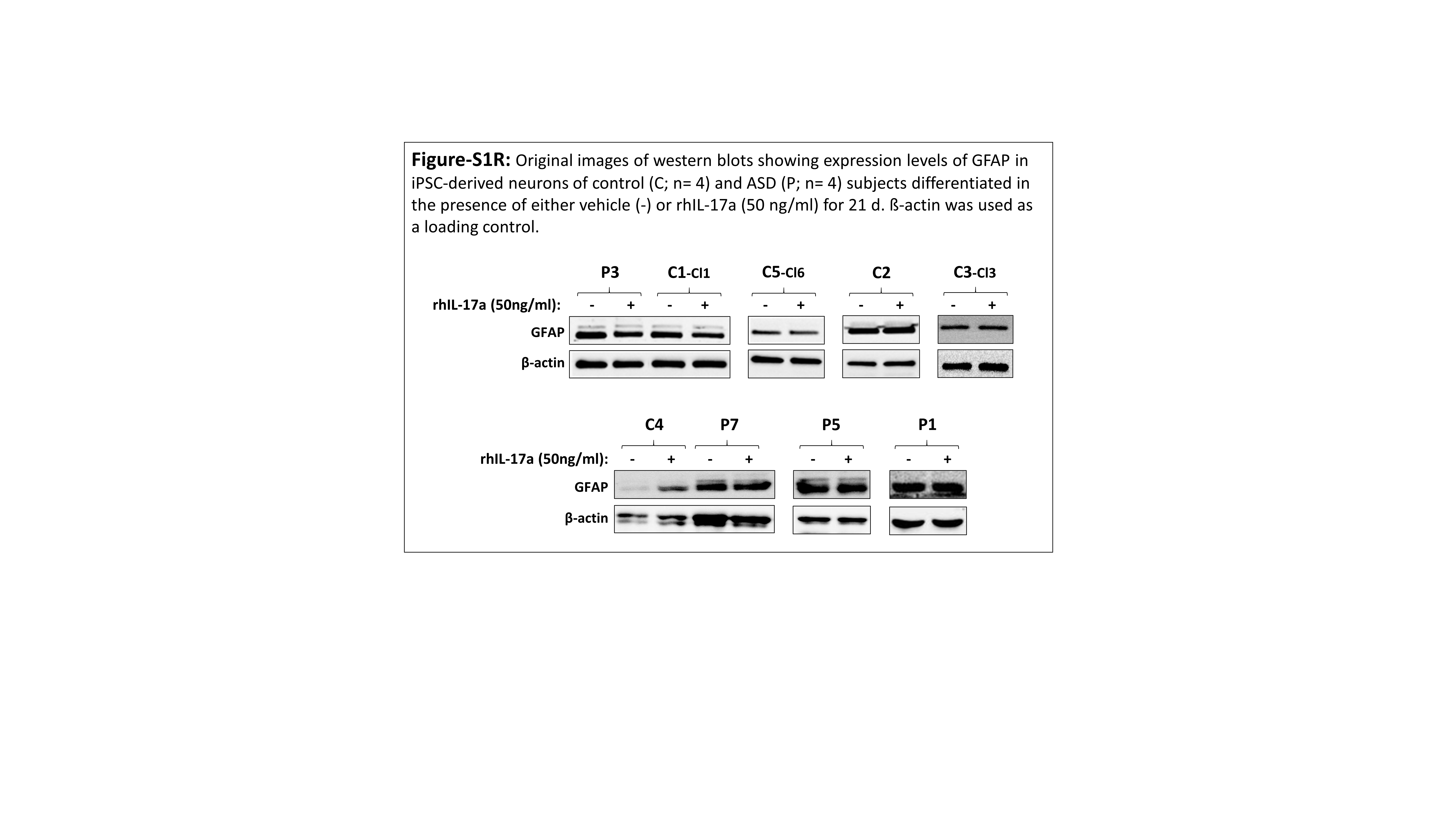

Supplement: Supplementary Figure 1 — Original western blot images used in this study. (A) Original images of western blots showing expression levels of IL17RA in iPSC-derived NPCs of control (C; n = 5) and ASD (P; n = 7) subjects. β-actin was used as a loading control. (B) Original images of western blots showing expression levels of IL17RA in iPSC-derived NPCs of control (C; n = 2–3) and ASD (P; n = 5) subjects after treatment with either vehicle (−) or rhIL-17a (10 and 50 ng/ml) for 48 h. β-actin was used as a loading control. (C) Original images of western blots showing expression levels of pERK1/2 in iPSC-derived NPCs of control (C; n = 4) and ASD (P; n = 6) subjects after treatment with either vehicle (−) or rhIL-17a (50 ng/ml). Total-ERK was used as a loading control. (D) Original images of western blots showing expression levels of pRPS6 in iPSC-derived NPCs of control (C; n = 3) and ASD (P; n = 4) subjects after treatment with either vehicle (−) or rhIL-17a (50 ng/ml). β-actin was used as a loading control. (E) Original images of western blots showing expression levels of pNF-kB65 in iPSC-derived astrocytes (used as positive control of pNF-kB expression) and in iPSC-derived NPCs of control (C; n = 2) and ASD (P; n = 1) subjects after treatment with either vehicle (−) or rhIL-17a (50 ng/ml). Total-NF-kB and β-actin were used as a loading controls. (F–M) Original images of western blots showing expression levels of markers for NPCs (SOX2 and Nestin), neurons (Synaptophysin-1, Synapsin-1, TUJ1, Homer-1 and MAP2) and astrocytes (GFAP) in iPSC-derived neurons of control (C; n = 3–4) and ASD (P; n = 3–6) subjects differentiated in the presence of either vehicle (−) or rhIL-17a (10 ng/ml) for 14 day. β-actin was used as a loading control. (N–R) Original images of western blots showing expression levels of markers for NPCs (SOX2), neurons (Synapsin-1, TUJ1, and MAP2) and astrocytes (GFAP) in iPSC-derived neurons of control (C; n = 3–5) and ASD (P; n = 3) subjects differentiated in the presence of eithe [file Image_10.TIFF]
